# Donor versus partner sperm in IVF/ICSI: risk of preeclampsia and obstetric outcomes in a prospective registry-linkage study

**DOI:** 10.1007/s10815-026-03935-w

**Published:** 2026-07-06

**Authors:** Berglind Kristjánsdóttir, Pelle Lindqvist, Frida Lundberg, Kenny A. Rodriguez-Wallberg

**Affiliations:** 1 Department of Reproductive Medicine, Karolinska University Hospital, Stockholm, Sweden; 2 Department of Clinical Science and Education, Karolinska Institutet, Stockholm, Sweden; 3 Department of Obstetrics and Gynaecology, Stockholm South General Hospital, Stockholm, Sweden; 4 Department of Medical Epidemiology and Biostatistics, Karolinska Institutet, Stockholm, Sweden; 5 Department of Oncology – Pathology, Karolinska Institutet, Stockholm, Sweden; 6 Department of Reproductive Medicine, Karolinska University Hospital, Stockholm, Sweden

**Keywords:** Preeclampsia, Sperm donation, Sperm freezing, Preconceptual exposure to semen, In vitro fertilisation, Pregnancy, Obstetric outcome

## Abstract

**Abstract:**

**Purpose:**

Immunological response to paternal antigens in semen and sperm has been implicated in the aetiology of preeclampsia. Although donor sperm conception has been associated with increased preeclampsia risk, evidence specifically regarding IVF with donor sperm remains limited. We aimed to assess whether pregnancies achieved via IVF with donor sperm are associated with a higher risk of preeclampsia or perinatal complications compared to IVF pregnancies using partner sperm.

**Methods:**

This prospective registry-linkage study included women undergoing IVF using donor versus partner sperm between 2009 and 2017 at the Reproductive Clinic of Karolinska University Hospital, Stockholm, Sweden. Data on demographics and IVF treatments were collected from the clinic’s database, while obstetric data were obtained from the Swedish Medical Birth Register by linking individual records across the two databases.

**Results:**

Compared with partner sperm, donor sperm was not associated with increased risk of preeclampsia. However, donor sperm was associated with an increased risk of 5-min Apgar score < 7 (OR 2.13, 1.21–3.78), but lower risks of preterm birth (OR 0.55, 0.35–0.86), preterm premature rupture of membranes (OR 0.35, 0.18–0.67), and low birth weight (OR 0.54, 0.33–0.88). Body mass index > 25 kg/m^2^ was associated with a higher rate of preeclampsia (aOR 2.20, 1.32–3.68).

**Conclusions:**

Our study does not support the hypothesis that sperm donation in IVF increases preeclampsia risk. However, donor sperm was associated with differences in obstetric and neonatal outcomes, suggesting that sperm source or related treatment characteristics may influence pregnancy outcomes beyond preeclampsia.

## Introduction

Preeclampsia remains a leading cause of maternal and perinatal morbidity and mortality. Identifying pregnancies at risk can decrease adverse events [1]. The aetiology remains elusive, but maladaptation of the mother’s immune system towards paternal antigens is a main hypothesis [2]. The observed risk for developing preeclampsia has been higher for nulliparous women and for multiparas after a change of paternity [3, 4]. These findings have suggested that an immunologic memory is established during the first pregnancy protecting against preeclampsia in subsequent pregnancies. Furthermore, a prior abortion or miscarriage fathered by the same partner is reported to halve the risk of preeclampsia in a subsequent pregnancy [5, 6]. In line with this, long-term sexual cohabitation before conceiving is also associated with decreased risk, while using barrier contraceptives seems to increase the risk [7, 8]. Furthermore, in pregnancies achieved with donor sperm, to which the mother is immunologically naïve, the risk for preeclampsia is reported to be increased [9]. This suggests that exposure to paternal semen tolerises the mother to feto-paternal alloantigens and that an immunological memory to partner’s antigens may be programmed at insemination [10]. It has been postulated that complete failure of this immunoregulation causes miscarriage whereas a partial failure increases the risk of preeclampsia [4, 11].

Prior systematic reviews have reported that pregnancies achieved with fertility treatments using sperm donation (SD) are associated with significantly increased risk of preeclampsia [9, 12]. However, in these analyses, different types of treatments were pooled together, and no distinction was made between pregnancies achieved with intrauterine insemination (IUI) and in vitro fertilisation (IVF) [9, 12]. A more recent large meta-analysis reported an increased risk for preeclampsia in pregnancies achieved with IUI using SD compared to partner sperm. Interestingly though, they found no difference in preeclampsia risk between the groups after IVF [13]. More recently, Liu and colleagues reported an overall increased risk of preeclampsia and pregnancy-induced hypertension (PIH) after pregnancies conceived with donor sperm compared with partner sperm, although the analysis included different conception methods [14]. The reasons for these apparently conflicting findings remain unclear.

In Sweden, SD-IVF treatments were introduced into the tax-funded healthcare system for heterosexual couples in 2003 and for women in same-sex relationships in 2005. In 2019, donor sperm IVF also became available at private IVF clinics. The selection of recipient couples for gamete donation follows a strict process. They must be deemed as fit for parenthood after a psychosocial and physical evaluation. The female age limit is 40 years and the partner’s is 56 years for the government-funded treatment. Couples who undergo IVF using their own gametes have similar health requirements and the same age limits as donor sperm.

This setting provides an opportunity to assess the effect of donor versus partner sperm within a population undergoing otherwise comparable IVF treatments. Although previous studies have reported an increased risk of preeclampsia after conception with donor sperm, much of the evidence is derived from cohorts combining different treatment modalities, particularly IUI and IVF. Furthermore, relatively few studies have been able to account for previous exposure to sperm from the same donor, limiting evaluation of the immunological naïvety hypothesis. By restricting our analysis to IVF/ICSI pregnancies and performing a subgroup analysis of women without previous treatment using sperm from the same donor, we sought to address these knowledge gaps.

The aim of this study was to evaluate whether the use of donor sperm increases the risk of preeclampsia in pregnancies achieved through IVF/ICSI and, by analysing a subgroup of women without previous exposure to sperm from the same donor, to directly examine the immunological naïvety hypothesis.

## Material and methods

This prospective study compared women who achieved pregnancy and live birth after IVF using donor sperm or partner sperm in a 1:2 ratio. All treatments were performed at the Reproductive Medicine Clinic of Karolinska University Hospital, Stockholm, Sweden, between January 1, 2009, and December 31, 2017. The couples went through psychosocial and clinical evaluation at the centre and fulfilled the required criteria for undergoing treatment. The sperm donors were between the ages of 25 and 46 years, passed psychosocial and health evaluation, demonstrated good sperm quality, had no family history of hereditary disease and chose to donate sperm for altruistic reasons to unrelated couples without any significant financial compensation. All the treated women in the cohort were healthy and younger than 40 years of age at the time of oocyte pick-up. The donor sperm was cryopreserved for at least 6 months before use. Matching (1:2) was based on the type of treatment (IVF or ICSI) and year of embryo transfer. Pregnancies were included independent of whether they were achieved by transfer of fresh or frozen–thawed embryos. All donor sperm was used after freezing and thawing, whereas only 5–7% of partner sperm samples were frozen–thawed. The Karolinska University Hospital serves a population of 2.2 million people and performs approximately 200 donor sperm IVF treatments annually [15]. Since 2003, IVF-physicians follow a national single-embryo transfer policy to reduce multiple conceptions [16].

### Ethical approval

The study was approved by the Regional Ethics Committee in Stockholm in 2011 (Dnr. 2011/1758–31/2) to allow prospective investigation of clinical outcomes of assisted reproduction and exempted specific signed consent for the study. The additional linking of individuals in the cohort to their data in the National Medical Birth Register was granted thereafter by the Swedish Ethics Authority, EPM (Dnr. 2023/01195–02).

### Data collection

Data on the IVF/ICSI treatments was extracted from the Reproductive Centre’s electronic database (LinneFiler®, Fertsoft AB, Uppsala, Sweden). The clinic enters patient data, including all relevant clinical and embryological data as well as data on pregnancy outcomes, prospectively into this database at the time of treatment. After birth, a detailed report is completed with information received from the parents, and a midwife controls the accuracy of the delivery data towards the regional obstetric register, ensuring that the database remains continuously updated, with ≥ 95% data completeness [17].

In Sweden, maternal healthcare is provided free of charge and all obstetric data for pregnancies and births are prospectively recorded into a standardised medical registry, the National Medical Birth Register (MBR) managed by the National Board of Health and Welfare. Data was collected via an online electronic medical registry, Obstetrix® (Cerner Sweden AB, Stockholm, Sweden), which records data on pregnancy, delivery and the postpartum period [18]. By using the patient’s unique personal identity number, data on the cohort were cross-matched with obstetric data from the MBR.

### Primary outcome

The primary outcome was preeclampsia, defined according to ICD-10 diagnoses recorded in the Medical Birth Register (MBR), including mild-to-moderate preeclampsia (O14.0), severe preeclampsia (O14.1), and HELLP syndrome (O14.2).

### Variables and other outcome measures

Demographic variables collected included: maternal age, parity, previous health conditions, smoking and body mass index (BMI). Data on the fertility treatment included whether IVF or ICSI was used, type of sperm (donor or partner sperm), the date of embryo transfer (ET) and embryo culture duration prior to transfer and if the embryo was fresh or frozen–thawed. Gestational length was calculated using the ET date and the delivery date. Preterm delivery was defined as occurring before 37 + 0 weeks of gestation. Obstetric outcome variables were extracted from the clinical database system based on their corresponding ICD-10 codes. Data were collected on preterm premature rupture of membranes (pPROM), placenta previa, placental abruption, and gestational diabetes. Mode of delivery (vaginal, instrumental or caesarean delivery) and severe postpartum haemorrhage (PPH) were recorded, with PPH defined as blood loss > 1000 ml within the first 2 h of delivery. Neonatal data including Apgar scores, gestational age at birth, newborn gender, birth weight, with low birth weight (LBW) defined as < 2500 g and small for gestational age (SGA) as birth weight below the 3rd percentile of expected weight for gestational age, based on a Swedish reference population [19].

### Statistical analyses

Background characteristics are presented as numbers and percentages or mean and standard deviations (SDs). Continuous variables, normally distributed, were compared using Student’s *t*-test; categorical variables were analysed with cross-tabulation with 95% confidence interval (CI) and chi-square test. Logistical regression analysis was performed with preeclampsia as dependent variable and other variables as independent (variables with a *p*-value < 0.1 in bivariate analysis). All tests were two sided with a significance level of 0.05. Statistical analyses were performed using IBM SPSS Statistics, Version 29.0.1.0.

## Results

During the study period, 500 women achieved pregnancy and live birth following either fresh embryo transfer (ET) or frozen ET (FET) after IVF/ICSI using donor sperm. A control group of 1000 women who conceived after IVF/ICSI, including ET and FET, with their partner’s sperm was selected. The control group was matched to the donor sperm group at a 1:2 ratio based on treatment type (IVF or ICSI) and calendar year of embryo transfer. During the study period, only 5–7% of partner sperm samples used for IVF/ICSI at our centre were frozen-thawed, compared with 100% of donor sperm samples.

Obstetric and neonatal outcome data were available for 1494 pregnancies and births, including 499 donor sperm cases and 995 partner sperm cases. Among the donor sperm group, 61.5% of couples were women in same-sex relationships. Demographic characteristics are summarised in Table 1.

Women in the partner sperm group were slightly younger, more often primiparous and more likely to have conceived after a fresh embryo transfer compared with women in the donor sperm group.

**Table 1:** Characteristics of patients who achieved pregnancies after IVF/ICSI using donor sperm or partner sperm

*n* %	Donor sperm		Partner sperm		*p*
	499	33%	995	67%	
Patient characteristics					
Age at ET					
Mean age ± SD	34.4	±3.6	33.2	±3.9	<.01
20–24 years	3	0.6%	17	1.7%	
25–29 years	45	9.0%	158	15.9%	
30–34 years	183	36.7%	419	42.1%	
35–39 years	241	48.3%	374	37.6%	
40 years	27	5.4%	27	2.7%	
Parity					<.01
0	316	63.3%	754	75.8%	
1	165	33.1%	208	20.9%	
≥2	18	3.6%	33	3.3%	
Body mass index					
Mean BMI ± SD	24.6	± 3.6	24.4	±4.2	.05
BMI <25 kg/m^2^	304	60.9%	633	63.6%	
BMI ≥25-<30 kg/m^2^	149	29.9%	243	24.4%	
BMI ≥30 kg/m^2^	46	9.2%	119	12.0%	
Smoking					.16
Yes	20	4.0%	57	5.7%	
Female partner	307	61.5%	0		
Treatment characteristics					
Year of ET					.99
2009-2011	108	21.6%	214	21.5%	
2012-2014	190	38.1%	383	38.5%	
2015-2017	201	40.3%	398	40.0%	
Type of treatment					.98
IVF	436	87.4%	869	87.3%	
ICSI	63	12.6%	126	12.7%	
Embryo transfer					.04
Fresh	276	55.3%	607	61.0%	
Frozen	223	44.7%	388	39.0%	

*IVF* in vitro fertilisation, *ICSI* intra-cytoplasmatic sperm injection, *ET* embryo transfer, *BMI* body mass index

Data is presented as mean ± standard deviation (SD) or *n* (%)

Table 2 compares obstetric and neonatal outcomes between the partner sperm group, the donor sperm group, and the general population. Incidence from the Swedish general population is given as a reference population. The vast majority of treatments resulted in singleton pregnancies with only 3% being twins. Preterm birth (9.1% vs. 5.2%), pPROM (6.0% vs. 2.2%) and low birth weight (7.8% vs. 4.4%) were all more common in pregnancies conceived with partner sperm than with donor sperm. These differences were consistent regardless of the partner’s sex and whether the transferred embryo was fresh or frozen–thawed (Table 4). The partner sperm group also had a lower average birth weight.

In contrast, newborns in the donor sperm group had a higher risk of having a 5-min Apgar score below 7 (5.0% vs. 2.4%). Rates of preeclampsia were similar in the partner and donor sperm groups (6.8% and 7.2%), but both were higher than the rate in the general population (2.2%).

**Table 2:** Obstetric and neonatal outcomes of the donor sperm vs. partner sperm groups. Swedish incidence is given as a reference

*n* (%)	**Donor sperm**		**Partner sperm**		OR (95% CI) *	**Reference**
	499	33%	995	67%	*p*	**Population**
Pregnancy conditions						
Multiple pregnancy	16	3.2%	31	3.1%	1.03 (0.56–1.90)	1.4%^a^
Gestational diabetes	2	0.4%	11	1.1%	0.36 (0.08–1.63)	0.8–2.4%^b^
Preeclampsia	36	7.2%	68	6.8%	1.06 (0.70–1.61)	2.7%^a^
Placenta previa	11	2.2%	22	2.2%	1.00 (0.48–2.07)	0.3%^a^
Placental abruption	5	1.0%	10	1.0%	1.00 (0.34–2.93)	0.4%^a^
PPH (> 1000 ml)	70	14.0%	135	13.6%	1.04 (0.76–1.42)	7.9%^a^
Retentio placentae	8	1.6%	13	1.3%	1.24 (0.51–3.01)	2.3%^a^
pPROM	11	2.2%	60	6.0%	**0.35 (0.18–0.67)**	
Female partner	8	2.3%			**0.37 (0.18–0.68)**	
Male partner	3	1.9%	60	6.0%	**0.31 (0.10–1.00)**	
Mode of delivery^d^						
Vaginal	287	57.5%	534	53.7%	1.17 (0.94–1.45)	75%^c^
Instrumental	55	11.0%	116	11.7%	0.94 (0.67–1.32)	8.0%^c^
Caesarean	157	31.5%	345	34.7%	0.86 (0.69–1.09)	17%^c^
Neonatal outcomes						
Female gender	273	54.7%	512	51.5%	0.88 (0.71–1.09)	49.6%^a^
Gestational age (days)	278.9	± 12.5	276.2	± 16.0	< 0.001	281 ± 13^a^
Preterm (< 37 w)	26	5.2%	91	9.1%	**0.55 (0.35–0.86)**	7.5%^a^
Birth weight (g)	3545	± 564	3371	± 595	< 0.001	3504^c^
LBW (< 2500 g)	22	4.4%	78	7.8%	**0.54 (0.33–0.88)**	4.2–4.5%^c^
SGA (< 2 SD for GA)	29	5.8%	55	5.5%	1.06 (0.66–1.68)	2.3–2.5%^c^
Low Apgar (< 7)	25	5.0%	24	2.4%	**2.13 (1.21–3.78)**	1.4–1.5%^c^

^a^Incidence in Sweden 1999–2002 (Thurn et al.) [20]

^b^Incidence in Sweden. The Pregnancy Register (Graviditetsregistret) yearly report 2016–2018

^c^Incidence in Sweden 2009–2017. The Medical Birth Register (Medicinska födelseregistret)

Data is presented as mean ± SD or *n* (%). Unadjusted odds ratios (OR) and 95% confidence intervals (CI) were calculated using the group of IVF/ICSI with partner sperm as reference. *Partner vs. donor sperm (ref)

The bolded data indicates that the 95% Confidence Interval (CI) for a ratio does not reach 1, indicating the finding as statistically significant

Abbreviations: *PPH* Postpartum haemorrhage > 1000 mL, *pPROM* preterm premature rupture of membranes, *IVF* in vitro fertilisation,* ICSI* intra-cytoplasmic sperm injection, *OR* odds ratio, *CI* confidence interval, *LBW* low birth weight < 2500 g, *low Apgar* < 7 after 5 min, *GA* gestational age, *SGA* small for gestational age, *SD* standard deviation

Table 3 presents the results of a logistic regression analysis on the risk of preeclampsia, with a subgroup analysis that restricts the donor sperm group to women who had not previously undergone IUI or IVF with sperm from the same donor. Neither the main analysis nor the subset analysis identified any difference in the risk based on parity, type of embryo (fresh or frozen), sperm source (donor or partner), treatment type (IVF or ICSI), smoking or maternal age. The only factor associated with an increased risk of preeclampsia was being overweight (BMI ≥ 25 kg/m^2^).

**Table 3:** Logistic regression analysis of the risk for preeclampsia and selected variables, crude and adjusted model* and an adjusted model** of a subset of the SD group including only women that had not gone through any previous treatment using sperm from the same donor

*n* (%)	No PE		PE		Crude model	Adjusted model*	Adjusted model**
	1390	93.0%	104	6.7%	OR (95% CI)	aOR (95% CI)	aOR (95% CI)
Parity							
Nullipara	1003	93.7%	67	6.3%	1.00 (ref)	1.00 (ref)	1.00 (ref)
Multipara	387	91.3%	37	8.7%	1.43 (0.94–2.17)	1.37 (0.90–2.11)	0.99 (0.57–1.71)
Type of embryo							
Fresh	828	93.8%	55	6.2%	1.00 (ref)	1.00 (ref)	1.00 (ref)
Frozen	562	92.0%	49	8.0%	1.31 (0.88–1.96)	1.27 (0.85–1.91)	1.26 (0.77–2.07)
Source of sperm							
Partner	927	93.2%	68	6.8%	1.00 (ref)	1.00 (ref)	1.00 (ref)
Donor	463	92.8%	36	7.2%	1.06 (0.70–1.61)	1.04 (0.68–1.60)	0.86 (0.36–2.08)
Type of treatment							
IVF	1211	93.8%	94	7.2%	1.00 (ref)	1.00 (ref)	1.00 (ref)
ICSI	179	94.7%	10	5.3%	0.72 (0.37–1.41)	0.74 (0.38–1.46)	0.78 (0.36–1.68)
Smoking							
No	1320	93.2%	97	6.8%	1.00 (ref)	1.00 (ref)	1.00 (ref)
Yes	70	90.0%	7	6.7%	1.36 (0.61–3.04)	1.22 (0.54–2.75)	1.13 (0.43–2.95)
Age at ET							
< 35 years	768	93.1%	57	6.9%	1.00 (ref)	1.00 (ref)	1.00 (ref)
≥ 35 years	622	93.0%	47	7.0%	1.02(0.68–1.52)	0.95 (0.63–1.43)	1.10 (0.68–1.79)
BMI							
< 25 kg/m^2^	1246	93.8%	83	6.2%	1.00 (ref)	1.00 (ref)	1.00 (ref)
≥ 25 kg/m^2^	144	87.3%	21	12.7%	**2.19 (1.32–3.64)**	**2.20 (1.32–3.68)**	**2.94 (1.68–5.17)**

*Adjusted for parity, type of embryo, source of sperm, type of treatment, year of ET, age at ET, BMI and smoking

**In the Subset analysis, the donor sperm group includes only women that had had no previous ART treatments using sperm from the same donor. *BMI*, body mass index; *ET*, embryo transfer; *IVF*, in vitro fertilisation; *ICSI*, intra-cytoplasmic sperm injection; *PE*, preeclampsia; *aOR*, adjusted odds ratio; *CI*, confidence interval

The bolded data indicates that the 95% Confidence Interval (CI) for a ratio does not reach 1, indicating the finding as statistically significant

Table 4 illustrates the results of a logistic regression analysis that shows that parity, type of embryo (fresh/frozen), type of treatment (IVF/ICSI), smoking and maternal age had no effect on the risk for LBW and pPROM. Multiple pregnancy increased the risk of pPROM and LBW, whereas donor sperm decreased the risk. Preeclampsia increased the risk of LBW while being overweight decreased the risk.

**Table 4:** Logistic regression analysis of the risk for LBW and pPROM and selected variables, adjusted model*

	LBW	pPROM
	aOR (95% CI)	aOR (95% CI)
Parity		
Nullipara	1.00 (ref)	1.00 (ref)
Multipara	0.85 (0.52–1.39)	1.29 (0.75–2.20)
Type of embryo		
Fresh	1.00 (ref)	1.00 (ref)
Frozen	0.87 (0.56–1.36)	0.73 (0.43–1.22)
Source of sperm		
Partner	1.00 (ref)	1.00 (ref)
Donor	**0.50 (0.30–0.83)**	**0.32 (0.16–0.62)**
Type of treatment		
ICSI	1.00 (ref)	1.00 (ref)
IVF	1.26 (0.65–2.46)	2.02 (0.84–4.85)
Smoking		
No	1.00 (ref)	1.00 (ref)
Yes	0.53 (0.16–1.78)	1.32 (0.50–3.49)
Multiple pregnancy		
No	1.00 (ref)	1.00 (ref)
Yes	**8.38 (4.07–17.26)**	**8.51 (3.79–19.13)**
PE		
No	1.00 (ref)	1.00 (ref)
Yes	**4.16 (2.35–7.37)**	1.12 (0.47–2.68)
BMI		
< 25 kg/m^2^	1.00 (ref)	1.00 (ref)
≥25 kg/m^2^	**0.37 (0.15–0.98)**	1.04 (0.49–2.21)
Age		
< 35 years	1.00 (ref)	1.00 (ref)
≥35 years	1.18 (0.77–1.81)	1.65 (1.00–2.71)

*Adjusted for parity, type of embryo, source of sperm, type of treatment, BMI, PE, Duplex, age and smoking

The bolded data indicates that the 95% Confidence Interval (CI) for a ratio does not reach 1, indicating the finding as statistically significant

*BMI* body mass index, *ET* embryo transfer, *IVF* in vitro fertilisation, *ICSI* intra-cytoplasmic sperm injection, *PE* preeclampsia, *aOR* adjusted odds ratio, *CI* confidence interval

## Discussion

Our analyses indicate that the use of donor sperm in IVF/ICSI does not increase the risk of preeclampsia or other adverse obstetric outcomes compared with partner sperm. A particular strength of this study is that it evaluated this question within a homogeneous IVF/ICSI population rather than combining different modes of conception, as has been the case in many previous studies. Furthermore, we were able to perform a subgroup analysis restricted to women with no previous treatment using sperm from the same donor, thereby allowing a more direct assessment of the immunological naïvety hypothesis. The absence of an increased risk of preeclampsia remained consistent in this subgroup, suggesting that lack of prior exposure to donor sperm antigens is not a major determinant of preeclampsia risk in IVF/ICSI pregnancies. The only factor associated with an increased risk of preeclampsia in this cohort was a BMI ≥ 25 kg/m^2^. The incidence of preeclampsia in both study groups was higher than that reported in the Swedish general population. This is consistent with previous meta-analyses showing that pregnancies achieved through IVF/ICSI have an increased risk of hypertensive disorders of pregnancy and preeclampsia compared with spontaneous conceptions, irrespective of sperm source [21]. Low birth weight (< 2500 g), pPROM and preterm birth were more frequent in the partner sperm group, whereas the risk of a 5-min Apgar score < 7 was significantly higher in the donor sperm group.

### Sperm donation and preeclampsia—the ART method matters

Immune priming by coitus has been demonstrated in mice, where exposure to seminal fluid modulates the uterine epithelial cells, promoting tolerance to paternal alloantigens and potentially facilitating implantation [22–24]. In humans, although the precise molecular mechanisms are less defined, seminal fluid induces a similar inflammation-like response in the cervix; whether this extends beyond the cervix remains unclear [25, 26].

The first suggestion that donor sperm may increase preeclampsia risk was made by Need and co-workers in 1983. They reported a preeclampsia incidence of 9.3% in 584 SD-IUI pregnancies, compared with the expected 0.5%–5%. They hypothesised that immunological naïvety contributed [27]. Subsequent studies have reported similar findings, showing increased preeclampsia risk in SD-IUI pregnancies compared with partner sperm IUI [28, 29]. In fact, numerous studies have demonstrated the importance of pre-conceptual exposure to semen as donor sperm, ICSI, short sexual relationships before conception and use of barrier methods of contraception have all been associated with increased risk of preeclampsia [30]. Exposure to partner’s semen, at the beginning of pregnancy, has even been shown to improve implantation and live birth rates after IVF [31].

Contrary to these findings, our study observed no increased risk of preeclampsia in donor sperm IVF pregnancies, despite lack of immune priming. However, our study results are in line with prior research studies specifically on IVF, rather than considering insemination or spontaneous conception. Kennedy and co-workers (2019) reported no difference in preeclampsia risk when comparing 1435 donor sperm IVF and 13,191 partner sperm IVF pregnancies [32]. Luke and team (2016) reached a similar conclusion in their analysis of data from a large register study, MOSART, finding no statistically significant difference in the risk of pregnancy-induced hypertension disorders (PIH) between SD-IVF and partner sperm IVF pregnancies [33]. Taken together, the available IVF-specific evidence does not support an increased risk of hypertensive disorders of pregnancy associated with donor sperm use.

### Could sperm cryopreservation alter antigenicity?

In Sweden, all donated sperm is cryopreserved for at least 6 months before being used, whereas only 5–7% of the partner sperm in our cohort was frozen. Cryopreservation alters sperm structure and function and can cause DNA fragmentation [34]. Cryopreservation may influence reproductive outcomes not only by damaging sperm function but also by altering immune-related gene activity. Mature sperm normally express HLA class I and II molecules, which help initiate an immune response in the female reproductive tract. This controlled immune activation is essential for establishing the immune tolerance needed for both semen exposure and early pregnancy. Elevated HLA class II expression has been reported in infertile men and has been associated with complications such as recurrent miscarriage and preeclampsia [11, 35]. Therefore, the increased HLA class II transcript levels observed after freezing may theoretically contribute to altered implantation potential when using cryopreserved sperm.

On the other hand, cryopreservation upregulates a distinct set of genes that seem to compensate for cryoinjury. These upregulated genes are mainly involved in antigen processing and presentation and can stimulate positive regulation of immune activation [35]. We therefore hypothesise that sperm cryopreservation may attenuate maternal immune responses to paternal antigens, thereby modifying the immunological interactions that occur during early pregnancy.

### Previous use of the same sperm donor

A common limitation of previous studies is the lack of information on whether women had undergone previous ART treatments using sperm from the same donor before achieving pregnancy. This could affect outcomes, as women with prior exposure, through repeated IUIs, early pregnancy loss or even a live birth, may have become immunologically familiar with the donor. Bartal and co-workers (2018) addressed this by comparing obstetric outcomes in 243 women who conceived after their first SD treatment (IUI or IVF) with 463 women who conceived after repeated treatments using the same donor’s sperm. They reported no difference in the risk of hypertensive disorders of pregnancy between the groups [36]. Our results are consistent with this: Prior treatment with sperm from the same donor was not associated with a reduced risk of adverse pregnancy outcomes.

### Sperm donation, LBW, preterm birth and Apgar scores

A few studies have examined whether the risk of LBW and preterm birth is influenced by the source of the sperm used in IVF/ICSI treatments. Two recent studies reported a significantly lower risk of LBW in fresh donor sperm IVF/ICSI cycles compared with cycles using partner sperm [37, 38], whereas another study found no difference in the risk of LBW between fresh donor sperm IVF and partner sperm IVF [39]. In line with our results, Pohjonen and co-workers (2022) reviewed data from five high-quality studies and concluded that the use of donor sperm in IVF/ICSI appears to decrease the risk of LBW [13]. None of these studies found differences in the incidence of preterm birth between the groups.

One possible explanation is that although the women were comparable regarding age, health and lifestyle factors, the underlying cause of infertility differs between the groups. Women in the donor sperm group were likely less affected by female-factor infertility, as 61.5% were in same-sex relationships and the other couples primarily underwent treatment because of male-factor infertility. A substantial body of literature links infertility itself with adverse obstetric and neonatal outcomes, regardless whether the conception occurred spontaneously or through ART [40]. Furthermore, recent studies have linked sperm defects and high sperm DNA fragmentation to adverse pregnancy and neonatal outcomes, including LBW, preterm birth and preeclampsia [41, 42]. Although speculative, it is possible that the quality of the donated sperm in our cohort was generally of higher quality than the partner sperm, even after cryopreservation, which might contribute to the differences we observed.

The increased risk of a 5-min Apgar score below 7 in the donor sperm group was unexpected, particularly as this group had lower rates of preterm birth and low birth weight. Given the relatively small number of affected infants, this finding should be interpreted with caution.

### Type of embryo and preeclampsia

Frozen–thawed embryo transfer has been associated with higher live birth rate and reduced risk of small for gestational age, LBW and preterm birth (< 37 weeks) [43]. However, contrary to our results, frozen ET has been reported to increase preeclampsia risk as reported by Roque and team in a large meta-analysis, confirming several previous observational studies [44].

### Sperm donation and pPROM

The foetal membranes act as a barrier for the foetus, providing it with mechanical and immunological protection. Preterm premature rupture of the membranes complicates up to 3% of pregnancies and precedes 30–40% of preterm births [45]. Known risk factors include infections, inflammation, oxidative stress, membrane stretching, intrauterine haemorrhage, iatrogenic procedures and lifestyle factors [46]. Women who conceive through ART, particularly after fresh ET cycles, are at higher risk [47]. However, data on pPROM in the context of sperm donation are limited.

In our study, the partner sperm group demonstrated a significantly higher risk of pPROM. Logistic regression analysis (Table 4) indicated that the higher frequency of fresh embryo transfers in the partner sperm group did not account for the increased risk. Factors related to female infertility or sexual practices may contribute to this observation.

## Strengths and limitations

A major strength of our study is that the data was prospectively collected into standardised healthcare databases with nearly 100% completeness. Data on the characteristics, pregnancy outcomes and perinatal data is near complete for all the women in the cohort. Several limitations should be acknowledged. First, there is a potential for selection bias, as this study could not be designed as a randomised controlled trial. Baseline differences between the groups, such as age, parity, embryo transfer type, infertility aetiology and lifestyle factors, including being in a same-sex relationship, may have influenced outcomes despite adjustments for known confounders. Donor sperm is obtained from rigorously screened donors with favourable sperm parameters, whereas partner sperm represents a more heterogeneous population, making it difficult to disentangle the effects of sperm source, sperm quality and underlying infertility characteristics. Moreover, all donor sperm was cryopreserved and thawed, which could affect the quality, whereas most of the partner sperm treatments used fresh sperm. Finally, information on whether sperm samples were fresh or frozen–thawed was generally not reported in previous studies, limiting comparison with our cohort.

## Conclusion

Preeclampsia is a multifactorial disorder. Previous research suggests that periconceptional exposure to paternal semen may reduce the risk and that the use of donor sperm increases the risk. However, IVF using donor sperm did not confer an increased risk of preeclampsia in our cohort. We speculate that this discrepancy between our findings and those reported in previous studies may be partially explained by sperm cryopreservation, which may alter the woman’s immunological response to paternal antigens. Another possible explanation relates to differences in the underlying infertility characteristics of the two groups. The primary indication for treatment in the donor sperm group was the absence of a male partner or severe male-factor infertility, whereas female-factor infertility may have been more common among women undergoing IVF/ICSI with partner sperm. As female infertility has been associated with adverse obstetric outcomes, any increase in preeclampsia risk associated with donor sperm may have been partially counterbalanced by a higher baseline risk in the partner sperm group. In addition, donor sperm was associated with lower risks of preterm birth, pPROM and low birth weight, but a higher risk of a 5-min Apgar score < 7, indicating that sperm source or related treatment characteristics may influence obstetric and neonatal outcomes beyond preeclampsia.

## Data Availability

No datasets were generated or analysed during the current study.

## Abbreviations

*aOR*: Adjusted odds ratio
*ART*: Assisted reproductive technology
*BMI*: Body mass index
*CI*: Confidence interval
*DNA*: Deoxyribonucleic acid
*ET*: Embryo transfer
*ICSI*: Intra-cytoplasmic sperm injection
*IVF*: In vitro fertilisation
*LBW*: Low birth weight (< 2500 g)
*OR*: Odds ratio
*PE*: Preeclampsia
*PPH*: Postpartum haemorrhage (> 1000 ml)
*pPROM*: Preterm premature rupture of membranes
*SD*: Sperm donation
*SD-IVF*: In vitro fertilisation using donated sperm
